# Characterization of the complete mitochondrial genome of *Glyptothorax minimaculatus* and phylogenetic studies of Sisoridae

**DOI:** 10.1080/23802359.2022.2093666

**Published:** 2022-07-28

**Authors:** Xiao Yang, Hailong Ge, Haoran Gu, Zhijian Wang

**Affiliations:** Key Laboratory of Freshwater Fish Reproduction and Development (Ministry of Education), Key Laboratory of Aquatic Science of Chongqing, School of Life Sciences, Southwest University, Chongqing, China

**Keywords:** *Glyptothorax minimaculatus*, mitochondrial genome, phylogenetic analysis, Sisoridae

## Abstract

The entire mitochondrial genome (mitogenome) of *Glyptothorax minimaculatus* was sequenced; it spanned 16,536 bp in length and contained 13 protein-coding genes (PCGs), 2 ribosomal RNAs, and 22 transfer RNA genes. A total of 37 genes formed a typical vertebrate mitochondrial gene arrangement. The phylogenetic tree of Sisoridae based on 13 PCGs was constructed and supported that *G. minimaculatus* was closely related to *Glyptothorax sinensis*, *Glyptothorax zanaensis*, *Glyptothorax longinema*, *Glyptothorax granosus* and *Glyptothorax lanceatus*. The mitogenome of *G. minimaculatus* described in this study provided molecular evidence for its current taxonomic status and laid a groundwork for further study concerning phylogenetics within Sisoridae.

*Glyptothorax minimaculatus* is a species in the family Sisoridae (Chen 2013) and is distributed in the Yingjiang River and Longchuan River in China. The description of this species has mainly focused on current morphology, and the genetic information of this species needs to be established. In this study, we sequenced and characterized the complete mitochondrial genome of *G. minimaculatus* and analyzed its phylogenetic position within Sisoridae. This research was expected to provide evidence for the current taxonomic status and investigate the phylogenetic relationship with other species in Sisoridae.

The specimen was obtained from the Longchuan River (Yunnan Province, China) with geographic coordinates of 25.024°N, 98.677°E and deposited at the zoological Museum of Southwest University under unique voucher number LJ-SCH-2-4-202001004 (the contact person is the corresponding author). The TIANamp genomic DNA Kit (Tiangen Biochemistry Technology Co., Ltd., China) was employed for total genomic DNA extraction from the muscle tissue. After amplification and purification, the DNA libraries were constructed and then sequenced by an Illumina NovaSeq 6000 sequencing platform. Sequenced fragments were assembled to procure the complete mitogenome by using GetOrganelle version 1.7.0+ (Jin et al. 2020) and NOVOPlasty version 4.2 (Dierckxsens et al. 2016). The entire mitogenome was annotated by using MITOS2 software (Bernt et al. 2013). All animal operations in this study were approved and supervised by the Committee of Laboratory Animal Experimentation at Southwest University (Chongqing Province, China) and strictly conformed to the guidelines (protocol number [2014]25) enacted by the committee.

The entire mitochondrial genome of *G. minimaculatus* spanned 16,536 bp in length and exhibited nucleotide contents of 31.17% A, 25.87% T, 15.41% G, and 27.54% C. The mitogenome formed a closed loop with a light (L) strand and a heavy (H) strand and comprised 13 protein-coding genes (PCGs, 11,414 bp in total), 2 ribosomal RNA genes (12S and 16S rRNA, 2,605 bp in total), and 22 transfer RNA (tRNA, 1,564 bp in total) genes. Except for *ND6* and 8 tRNAs (Ala, Asn, Cys, Tyr, Ser2, Glu, Pro, Gln), which were located on the L-strand, the residuals were distributed on the H-strand (Miya and Nishida 2000). Furthermore, the original replication on the L-strand and the control region (D-loop) on the H-strand were predicted.

In 13 PCGs, ATG was identified as the canonical start codon for 12 PCGs, with the particular case that the *COI* gene applied the GTG as its start codon. The use of stop codons was varied as follows: TAA: *COI*, *ATP6*, *ATP8*, *ND1*, *ND4L* and *ND5*; TGA: *ND2*, *ND3* and *ND6*; TA-: *COIII*; T-: *COII*, *Cytb and ND4*. The incomplete stop codons of TA- and T- are ordinarily present in fish mitochondrial genes (Lee et al. 2019). Twenty-two tRNAs distributed between rRNAs and PCGs were detected based on their respective anticodon sequences. The 12S and 16S rRNAs clamped by *tRNA^Phe^* and *tRNA^Leu(TAA)^* and interposed by *tRNA^Val^* were 957 bp and 1,648 bp, respectively.

A phylogenetic tree based on concatenated sequences of 13 PCGs in 20 fish species was constructed by using the neighbour-joining method with 1000 bootstrap replicates in the software MEGA 11 (Figure 1) (Tamura et al. 2021). *G. minimaculatus* was clustered into the *Glyptothorax* branch and closely related to *Glyptothorax sinensis*, *Glyptothorax zanaensis*, *Glyptothorax longinema*, *Glyptothorax granosus* and *Glyptothorax lanceatus*, with bootstrap probabilities of 100%. The position of *G. minimaculatus* in the NJ tree supported its current taxonomic status based on morphological features and clearly indicated that it is a valid species.

**Figure 1.**
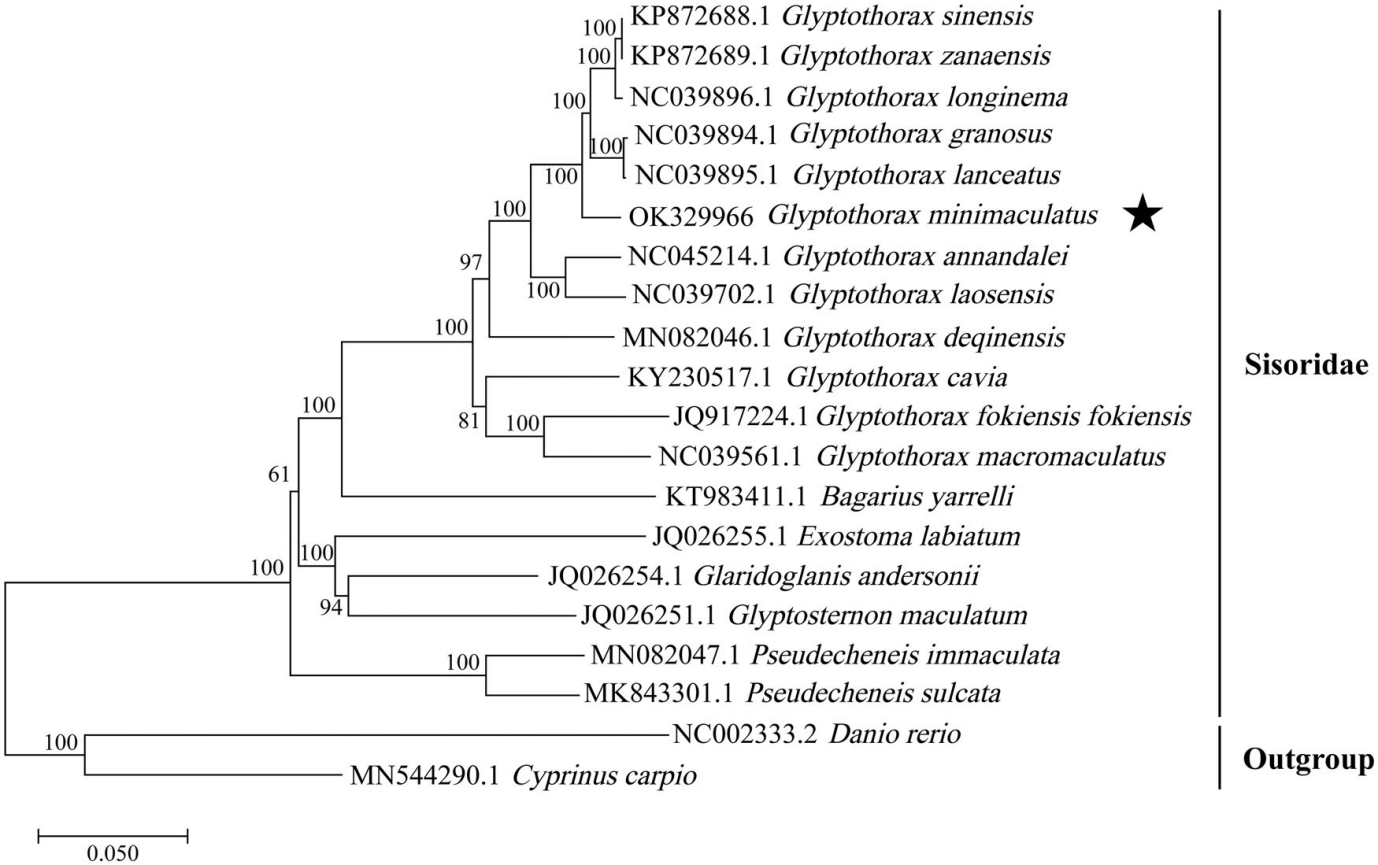
Neighbour-joining phylogenetic tree (bootstrap 1000) based on 13 mitochondrial protein-coding genes from 20 species. *Glyptothorax minimaculatus* is highlighted by a ‘black pentacle,’ and bootstrap support values are shown on the nodes.

## Data Availability

The genome sequence data that support the findings of this study are openly available in GenBank of NCBI under the accession no. OK329966 (https://www.ncbi.nlm.nih.gov/nuccore/OK329966). The associated BioProject, SRA, and Bio-Sample numbers are PRJNA786366, SRR17200913, and SAMN23669246, respectively.

## References

[CIT0001] Bernt M, Donath A, Jühling F, Externbrink F, Florentz C, Fritzsch G, Pütz J, Middendorf M, Stadler PF. 2013. MITOS: improved de novo metazoan mitochondrial genome annotation. Mol Phylogenet Evol. 69(2):313–319. 22982435 10.1016/j.ympev.2012.08.023

[CIT0002] Chen XY. 2013. Checklist of fishes of Yunnan. Dongwuxue Yanjiu. 34(4):281–343. 23913883 10.11813/j.issn.0254-5853.2013.4.0281

[CIT0003] Dierckxsens N, Mardulyn P, Smits G. 2016. NOVOPlasty: de novo assembly of organelle genomes from whole genome data. Nucleic Acids Res. 45(4):gkw955. 10.1093/nar/gkw955 PMC5389512 28204566

[CIT0004] Jin J-J, Yu W-B, Yang J-B, Song Y, dePamphilis CW, Yi T-S, Li D-Z. 2020. GetOrganelle: a fast and versatile toolkit for accurate de novo assembly of organelle genomes. Genome Biol. 21(1):1–31. 10.1186/s13059-020-02154-5 PMC7488116 32912315

[CIT0005] Lee S-H, Kang C-B, Shin M-H, Lee S-H, Yoon M, Kim HJ. 2019. The complete mitochondrial genome of a marbled eelpout *Lycodes raridens* (Perciformes: Zoarcidae). Mitochondrial DNA B Resour. 4(2):4043–4044. 33366309 10.1080/23802359.2019.1688698 PMC7707741

[CIT0006] Miya M, Nishida M. 2000. Use of mitogenomic information in teleostean molecular phylogenetics: a tree-based exploration under the maximum-parsimony optimality criterion. Mol Phylogenet Evol. 17(3):437–455. 11133198 10.1006/mpev.2000.0839

[CIT0007] Tamura K, Stecher G, Kumar S. 2021. MEGA11: molecular evolutionary genetics analysis version 11. Mol Biol Evol. 38(7):3022–3027. 33892491 10.1093/molbev/msab120 PMC8233496

